# s-Triazine: A Privileged Structure for Drug Discovery and Bioconjugation

**DOI:** 10.3390/molecules26040864

**Published:** 2021-02-06

**Authors:** Anamika Sharma, Rotimi Sheyi, Beatriz G. de la Torre, Ayman El-Faham, Fernando Albericio

**Affiliations:** 1 Peptide Science Laboratory, School of Chemistry and Physics, University of KwaZulu-Natal, Durban 4001, South Africa; anamika.aug14@gmail.com (A.S.); ebenrotex4fun@gmail.com (R.S.); garciadelatorreb@ukzn.ac.za (B.G.d.l.T.); 2 KwaZulu-Natal Research Innovation and Sequencing Platform (KRISP), School of Laboratory Medicine and Medical Sciences, College of Health Sciences, University of KwaZulu-Natal, Durban 4041, South Africa; 3 Department of Chemistry, College of Science, King Saud University, P.O. Box 2455, Riyadh 11451, Saudi Arabia; 4 Chemistry Department, Faculty of Science, Alexandria University, P.O. Box 426, Ibrahimia, Alexandria 12321, Egypt; 5 Institute for Advanced Chemistry of Catalonia (IQAC-CSIC), 08034 Barcelona, Spain; 6 CIBER-BBN (Networking Centre on Bioengineering, Biomaterials and Nanomedicine) and Department of Organic Chemistry, University of Barcelona, 08028 Barcelona, Spain

**Keywords:** s-triazine, nucleophiles, azide, orthogonal chemoselectivity

## Abstract

This review provides an overview of the broad applicability of s-triazine. Our many years working with this intriguing moiety allow us to discuss its wide activity spectrum (inhibition against MAO-A and -B, anticancer/antiproliferative and antimicrobial activity, antibacterial activity against MDR clinical isolates, antileishmanial agent, and use as drug nano delivery system). Most of the compounds addressed in our studies and those performed by other groups contain only *N*-substitution. Exploiting the concept of orthogonal chemoselectivity, first described by our group, we have successfully incorporated different nucleophiles in different orders into s-triazine core for application in peptides/proteins at a temperature compatible with biological systems.

## 1. Introduction

**Figure 1:**
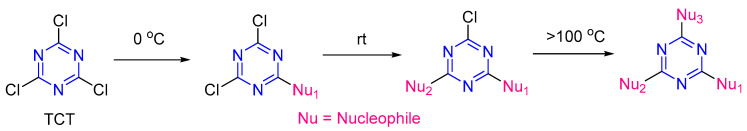
2,4,6-Trichloro-1,3,5-triazine (TCT) reactivity toward the nucleophilic substitution reaction.

**Figure 2:**
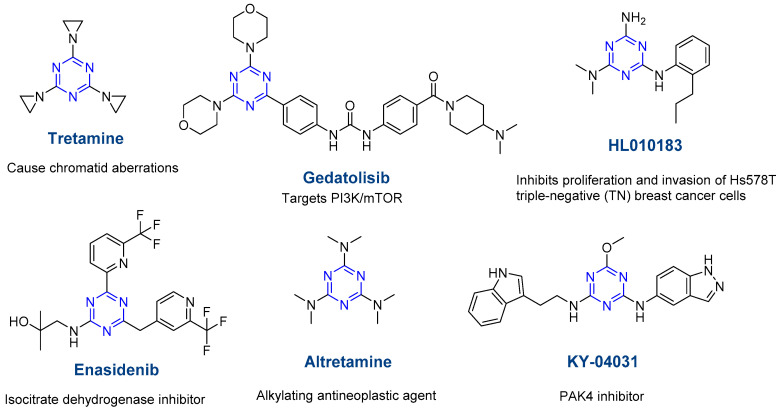
Some commercial drugs bearing the s-triazine ring.

1,3,5-Triazine as s-triazine is an extensively studied privileged structure in medicinal chemistry [1,2,3,4,5]. 2,4,6-Trichloro-1,3,5-triazine (TCT) is the starting core to afford several s-triazine derivatives, taking advantage of its low cost and easy manipulation of three independent, readily tunable ring positions, which facilitates the sequential nucleophilic substitutions reactions with almost all types of nucleophiles (S, O and N) [6,7,8,9,10,11,12]. Figure 1 shows the typical order of nucleophile incorporation onto TCT. The incorporation of the first nucleophile can be performed at 0–5 °C, while the second one requires room temperature, and the third heating or reflux [13,14,15]. The reactivity of the s-triazine ring decreases upon substitution with different nucleophiles due to a gain of π-orbital electron density, thereby reducing the effectiveness of further nucleophilic substitution reactions under similar conditions and thus requiring a higher temperature for further substitutions [8,16]. 

s-Triazine is extensively studied because of its wide applications in biological systems as an antibacterial, antiviral, anticancer, and antifungal agent, etc. [1,5,17,18]. Several commercial drugs contain the s-triazine core (Figure 2) [3,5]. Apart from their application as pharmacophores and in medicinal chemistry, s-triazine derivatives are useful as organic reagents, energetics, and new materials (dendrimers, supra-molecular aggregates, etc.) [4,12,19,20].

Taking advantage of the unique reactivity shown by TCT, we have synthesized several s-triazine derivatives and evaluated their activity for several biological targets, which will be discussed in subsequent sections [13,14,21,22,23,24,25,26,27,28]. TCT has been further explored as a linker that encompasses two key chemical concepts, namely orthogonality and chemoselectivity [8,16,29]. This review compiles all these results on TCT and broadly covers the following three sections: synthetic strategy; biological activity; and orthogonal chemoselectivity.

## 2. Synthesis of s-Triazine Derivatives

**Scheme 1:**
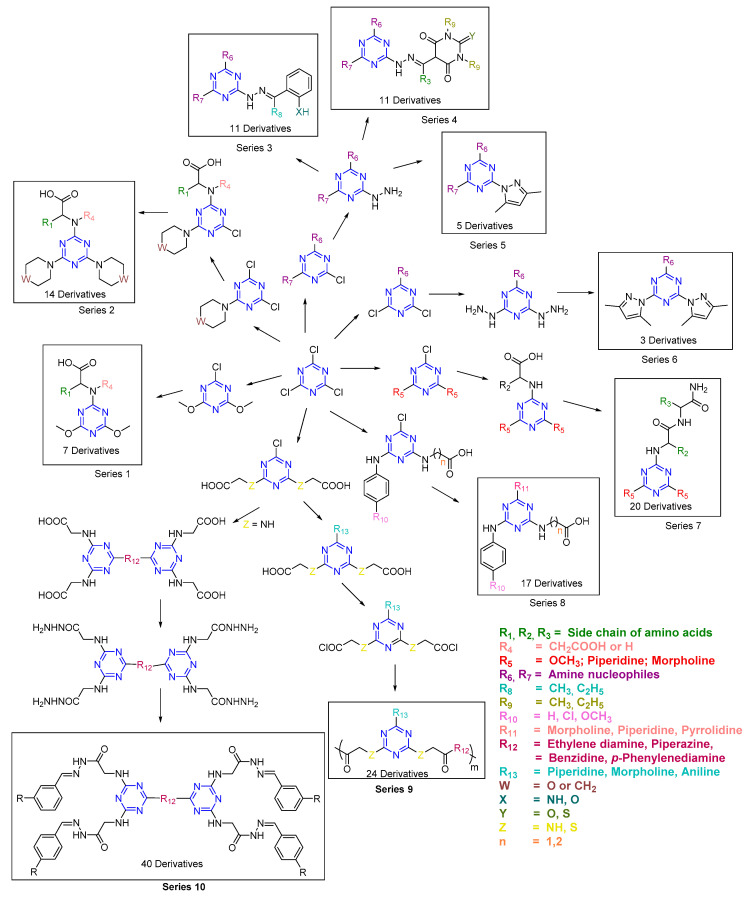
Scheme for the synthesis of s-triazine derivatives.

Several derivatives were synthesized via different routes, resulting in eight series, as shown in Scheme 1. The replacement of the first and second Cl atoms was achieved in the presence of a base at 0 °C and room temperature (rt), respectively. Different nucleophiles like piperidine, morpholine, benzylamine, and methanol (MeOH) were used for the replacement of the first and second Cl atoms in the presence of bases such as triethylamine (TEA), diisopropylethylamine (DIEA), Na_2_CO_3_, etc. Series 1 was synthesized using dimethoxy-substituted s-triazine [21]. In this series, a solution of dimethoxy-substituted s-triazine and TEA in dioxane was reacted until a white suspension formed. A solution of amino acid and TEA in dioxane-H_2_O (1:1) was added to form a clear solution. The reaction was stirred overnight at rt followed by neutralization with 1N HCl to yield a white solid, which afforded the desired products (seven derivatives) upon filtration and drying. In Series 2, a protocol similar to that described above was followed for the second substitution in piperidine/morpholine—substituted using monosubstituted dichlorotriazine (m-DCT). After stirring the reaction overnight at rt, a solution of piperidine/morpholine and TEA was added to replace the third Cl atom. The reaction mixture was heated at 75–80 °C for 5 h and then neutralized with 5% citric acid/1N HCl, resulting in precipitation. Filtration and drying afforded the desired products (14 derivatives) [21].

Hydrazone derivatives bearing a s-triazine core (Series 3 and 4) were synthesized [13,15,24]. Disubstituted-monochloro triazines (d-MCTs) were synthesized using piperidine and morpholine as nucleophiles. NaHCO_3_ was used as base. The first Cl atom replacement was performed at 0 °C for 2 h followed by in situ replacement of the second Cl atom at rt overnight. The product was precipitated upon removal of the solvent. After filtration and drying, the product was reacted with hydrazine using ethanol as solvent. The reaction was sonicated at 60 °C for 1 h. Ethanol was removed, and the desired product (hydrazinyl trisubstituted s-triazine) was precipitated using excess diethyl ether. A Schiff base was synthesized using two methods (one method under reflux and the other method in an Erlenmeyer flask). To this end, 2–3 drops of acetic acid, followed by hydrazinyl trisubstituted s-triazine, were added to a solution of 2-substituted acetophenone (for Series 3) or pyrimidinetrione/thiopyrimidinetrione (for Series 4) in ethanol. The reaction mixture was refluxed for 3 h (in the other case, at 40 °C for 1 h). In both cases, after reaction, ethanol was removed, resulting in precipitation. The reaction mixture was then filtered and dried. Recrystallization (ethyl acetate or ethanol) afforded the desired products (Series 3 and 4).

Pyrazole derivatives (Series 5 and 6) were synthesized by Method A and Method B [14]. Initially, m-DCTs and d-MCTs derivatives bearing piperidine, morpholine, or benzyl amine were prepared as per the procedure explained above. m-DCTs/d-MCTs were reacted with hydrazine hydrate to afford di/mono-hydrazinyl trisubstituted s-triazine. In Method A, this compound was dissolved in DMF and acetylacetone was then added. TEA was added and the reaction was refluxed for 6–8 h. Ice-cooled H_2_O was added to precipitate the desired products, which were obtained by filtration, drying, and recrystallizing using ethanol (Series 5 and 6). In Method B, acetylacetone was added dropwise to a solution of mono/di-hydrazinyl trisubstituted s-triazine in HClO_4_. The reaction was stirred at rt overnight. The reaction mixture was neutralized with K_2_CO_3_ to form a precipitate. Filtration and drying afforded the desired products (Series 5 and 6) as above. Method B afforded better yields than Method A.

Amino acid derivatives of s-triazine (Series 7) were prepared as explained for Series 1 [22]. 1-[Bis(dimethylamino)methylene]-1H-1,2,3-triazolo[4,5-b]pyridinium 3-oxide hexafluorophosphate (HATU) was used as coupling reagent in the presence of DIEA as base. A solution of amino acid ester in DIEA and DMF was added to the stirring solution of the amino acid derivative of triazine. The reaction was stirred overnight at rt. The reaction mixture was diluted with ethyl acetate and extracted using 5% citric acid, sat. NaHCO_3_, and sat. NaCl. The organic compound was collected, dried using MgSO_4_, filtered, and concentrated to afford the desired products (Series 7).

Disubstituted s-triazine derivatives (Series 8) were synthesized as explained earlier using amino acids/substituted anilines and aq. NaHCO_3_ as base (first Cl atom replacement at 0 °C for 2 h and second Cl atom at rt overnight). The third nucleophile (morpholine, piperidine, and pyrrolidine) was reacted with disubstituted s-triazine in the presence of DIEA as base and THF as solvent using three approaches, namely conventional heating (reflux 20–24 h), microwave (12–15 min at 70 °C), and ultrasonication (1.5 h at rt). A total of 17 derivatives were synthesized using all three approaches (Series 8). The last two approaches outperformed conventional heating in terms of yield and purity [30].

Diamino acid derivatives of s-triazine were prepared as explained above using TCT and two equiv. of glycine/thioglycolic acid with Na_2_CO_3_ as base and acetone-H_2_O as solvent. The reaction was stirred at rt for 24 h to afford the desired products [27,28], which were further reacted with different amines (piperidine, morpholine, or aniline) in the presence of Na_2_CO_3_ as base in dioxane and under reflux conditions for 24 h. The product was then reacted with thionyl chloride to regenerate the diacid chloride, which was then reacted with diamine (ethylene diamine, benzidine, piperazine, or *p*-phenylenediamine) in TEA and DMF to afford the desired polyamides (Series 9, Scheme 1) [27].

The diamino acid derivatives were further reacted with diamine as mentioned above in dioxane-H_2_O as solvent and Na_2_CO_3_ as base under reflux for 24 h to afford tetracarboxylic acid derivatives. The hydrazide derivatives were synthesized by reacting the tetracarboxylic acid derivatives with MeOH in the presence of H_2_SO_4_ as catalyst, followed by a reaction with hydrazine hydrate. Upon the reaction with different aldehydes in dioxane-H_2_O (reflux 3 h), these derivatives afforded the desired Schiff base derivatives (Series 10, Scheme 1).

## 3. Biological Evaluation

### 3.1. MAO-A and -B Inhibitors

**Figure 3:**
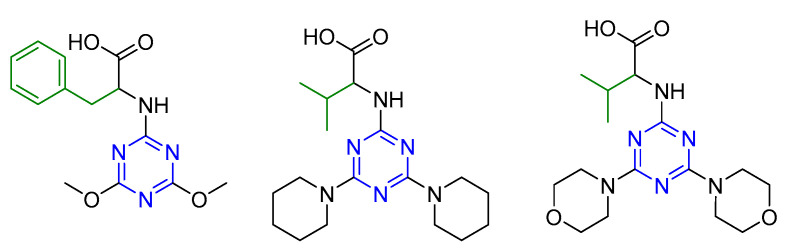
Potential compounds as inhibitors of monoamine oxidase A and B (MAO-A and B).

The biological activity of all the synthesized derivatives was evaluated. Series 1 and 2 were examined as inhibitors of monoamine oxidase A and B (MAO-A and -B). Of the 21 derivatives synthesized, the inhibitory effect of three was comparable to the reference compound clorgyline, and they showed greater selectivity toward MAO-A than MAO-B and with no significant toxicity (Figure 3) [21].

### 3.2. Antiproliferative/Anticancer Activity

**Figure 4:**
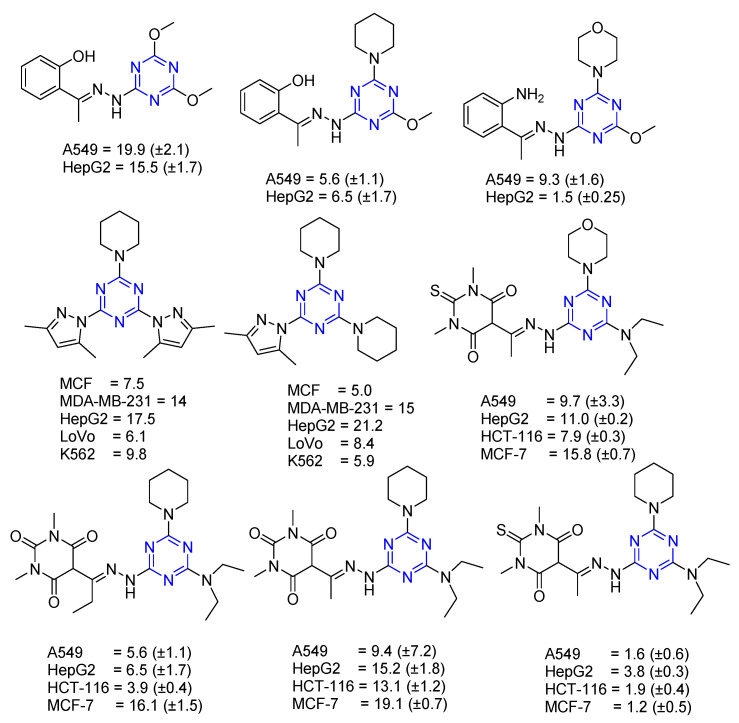
Compounds showing antiproliferative/anticancer activity (IC_50_).

Series 3 and 4 (Schiff base analogs) [13,24] and Series 6 (Pyrazole) [23] were evaluated for anticancer activity against Lung carcinoma (A549), Hepatocellular carcinoma (HepG2), Adenocarcinoma (MCF-7), Human breast cancer (MCF, MDA-MB-231), Human colorectal carcinoma (LoVo, HCT-116), and Human leukemia (K562). Out of all the 34 derivatives, Figure 4 shows the derivatives possessing Schiff base analogs, which showed higher antiproliferative/anticancer activity than pyrazole derivatives (Figure 4). The presence of two pyrazoles on the s-triazine core had a negative impact on bioactivity. Of the Schiff base derivatives, pyrimidinetrione/thiopyrimidinetrione appeared to be the best choice for A549 and HepG2 cell lines. s-Triazine bearing thiopyrimidinetrione with piperidine enhanced the activity compared to morpholine [13,23,24].

### 3.3. Antimicrobial Activity

**Figure 5:**
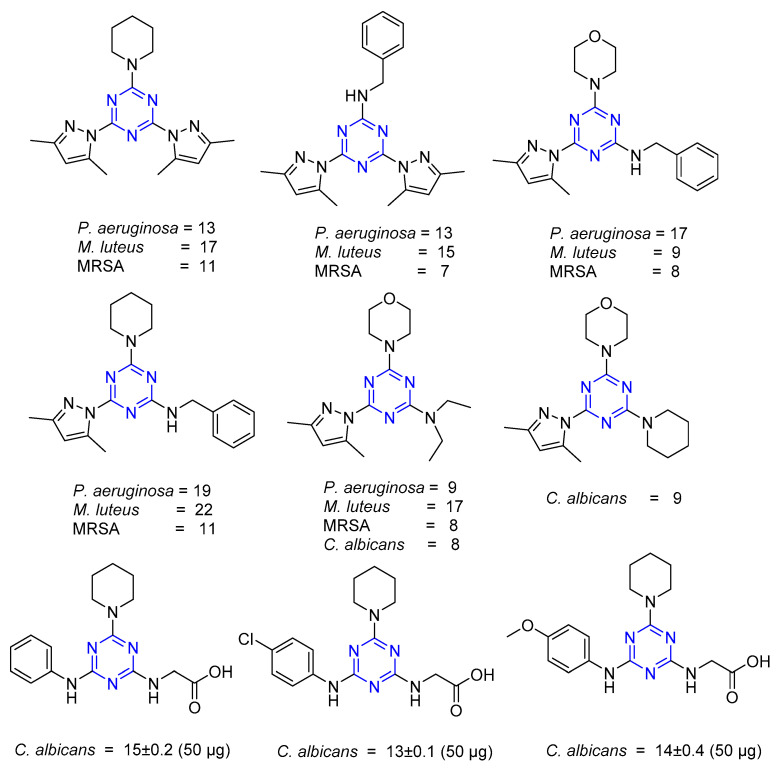
Compounds with antimicrobial activity (as zone of inhibition in mm).

Pyrazole derivatives (Series 5 and 6) and substituted aniline derivatives (Series 8) were evaluated for antimicrobial activity (measured as zone of inhibition in mm) [14,30]. Figure 5 shows the structures of all the active analogs. The activity of the derivatives against cultures of Gram-negative bacteria (*E. coli* ATCC 25922, *P. aeruginosa* ATCC 75853, *S. typhimurium* ATCC 14028), Gram-positive bacteria (*M. luteus* ATCC 10240, Methicillin-resistant *Staphylococcus aureus* (MRSA) ATCC 43300, *S. epidermidis* ATCC 12228), and *C. albicans* ATCC 10145 and 60193 (fungi) was tested. Two pyrazole rings incorporated in the s-triazine ring are detrimental to the antimicrobial activity compared to one ring. Of all the derivatives, s-triazine with pyrazole, benzylamine, and piperidine have been found to be most active. The presence of the morpholine ring along with the secondary amine is relevant for antifungal activity [14]. The presence of piperidine in Series 8 appeared to be important for antifungal activity. Electron-withdrawing groups like Cl enhanced antifungal activity compared to their methoxy counterparts [30].

**Figure 6:**
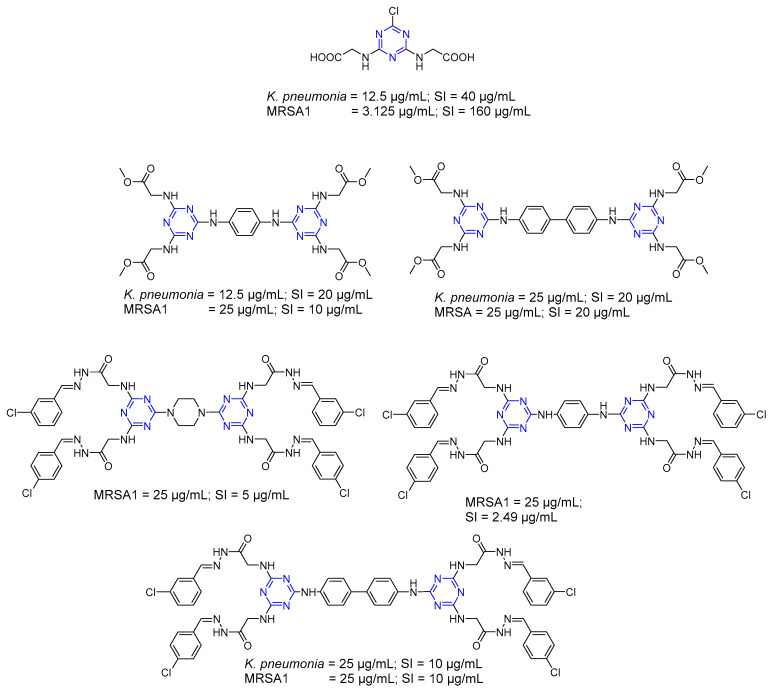
Minimum Inhibitory Concentration (MIC) and selectivity index (SI) of compounds showing activity against *K. pneumonia* and MRSA1.

Dimeric s-triazine hydrazide and its Schiff base derivatives (Series 9) were screened in vitro for antimicrobial activity against *S. aureus* (ATCC 19433), *E. coli* (ATCC 25922), and *C. albicans* as a yeast-like fungus [28]. The derivatives showed good antibacterial activity (Figure 6) but no antifungal activity. The active compounds were further screened for antibacterial activity against multidrug-resistant (MDR) clinical isolates. The results showed a promising inhibition efficiency, comparable to the standard drug ampicillin trihydrate. The active compounds also showed a high selectivity index toward antimicrobial activity compared to mammalian cells, with a good safety profile. Electron-withdrawing groups were crucial for enhancing antibacterial activity [28]. Moreover, an aliphatic core appeared to have a detrimental effect on the activity compared to an aromatic core [28].

The feasibility of s-triazine polyamides containing Gly and thioglycolic acid (Series 10) as drug nano delivery systems was examined. The nanoparticles (NPs) were loaded with celecoxib (CXB), an anti-inflammatory drug with a promising anti-cancer effect. The NPs showed high entrapment efficiency levels (62.3–99.8%) with drug loading between 1.58% and 4.19%. After 48 h, 46.90, 64.20, 57.81, 53.95, and 49.43% of CXB were released from polymeric NPs 26, 43, 44, 45, and 46, respectively, thereby demonstrating a sustained drug release profile. Notably, free CXB and CXB-loaded polymeric NPs CXB-43, CXB-45, and CXB-46 caused a considerable reduction in cell viability in a dose-dependent manner. Moreover, NPs were cytotoxic against breast cancer cells (MCF-7). Overall, these NPs hold great promise as drug delivery systems and are expected to accumulate significantly in tumor tissues via the EPR effect, thereby maximizing the antitumor efficacy of drugs and reducing systemic toxicity [27].

### 3.4. Antileishmanial Activity

**Figure 7:**
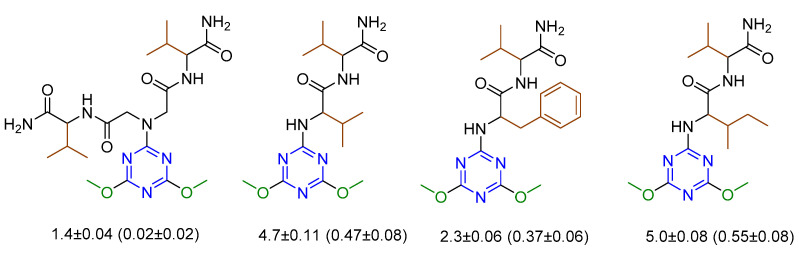
Compounds active against antileishmanial activity. The values are measured as IC_50_ in µM for anti-promastigote (anti-amastigote) activity.

Series 7 (20 derivatives) comprising the hybrid molecules of the 1,3,5-triazine moiety and short hydrophobic peptides were evaluated against *L. aethiopica* using miltefosine and amphotericin B as reference drugs [22]. The in vitro anti-promastigote activity of all the derivatives was studied. Four compounds were found to be active. The dimethoxy derivatives were more active than the corresponding dipiperidine and dimorpholine derivatives. The derivative with Gly-Val dipeptide was five times more active than miltefosine. These four derivatives were further tested for in vitro anti-amastigote and cytotoxicity followed by in vivo anti-toxicity (Figure 7). The compounds showed low toxicity. A combination of cytotoxicity and antileishmanial activities showed greater selectivity toward the protozoa (*L. aethiopica*) than mammalian cells, indicating a safe toxicity profile.

## 4. Orthogonal Chemoselectivity

The uniqueness of TCT undergoing a sequential nucleophilic substitution reaction has been exploited to explain orthogonal chemoselectivity. Orthogonality and chemoselectivity are two interchangeable words widely used in bioconjugation, although the former is a subset of the latter. Orthogonality was first introduced by Barany and Merrifield in 1977 [31] and demonstrated by Barany and Albericio in 1985 as “Orthogonal protecting groups”, which can be successfully removed in any order by various chemical mechanisms and in the presence of other protecting groups [32]. At the same time and in parallel, in 1983, Trost coined the term “Chemoselectivity” to describe the ability of a chemical reagent to discriminate among reactive sites [33]. The fusion of these two concepts, “Orthogonal Chemoselectivity”, was defined by our group as a “discrimination between reactive sites in any order” [8].

### 4.1. Reactivity of TCT with Nucleophiles

**Scheme 2:**
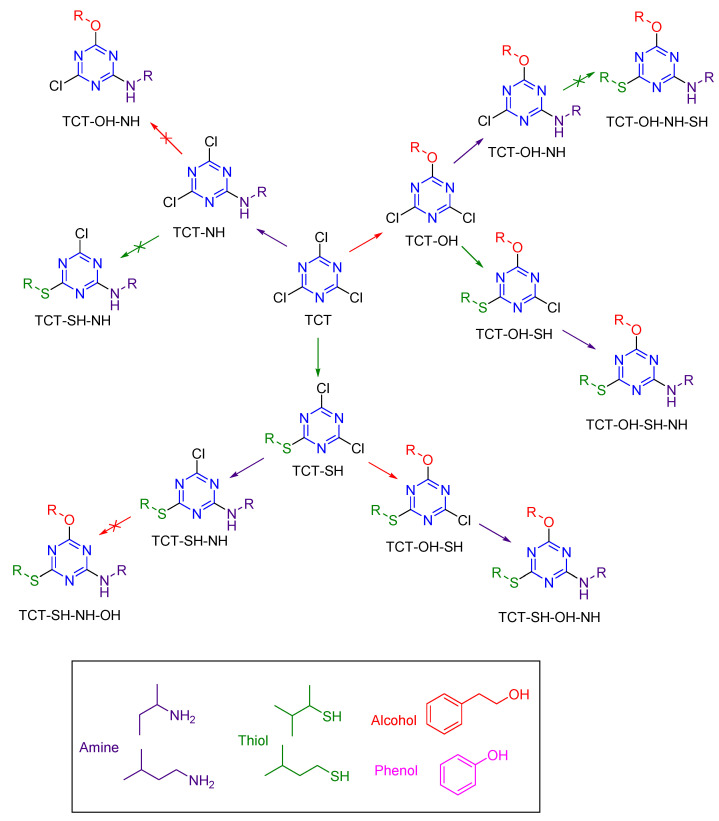
Reactions attempted on TCT with nucleophiles (NH, SH, and OH/pOH) and the preferential order of incorporation was found to be first alcohol/phenol, second thiol, and third amine.

The concept of Orthogonal Chemoselectivity using TCT as the core linker using amine (NH), thiol (SH), and alcohol/phenol (OH/pOH) as nucleophiles (mimicking amino acid side chains present in proteins) has been demonstrated [8,9]. Scheme 2 shows the series of reactions attempted to explain the order of incorporation.

**Figure 8:**
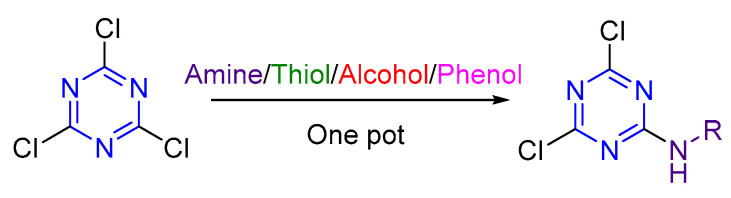
Competitive reaction of TCT with all nucleophiles in one pot.

TCT was reacted in parallel with NH, SH, and OH in the presence of DIEA at 0 °C to form m-DCTs. Since all the reactions to form m-DCTs (TCT-NH, TCT-OH, and TCT-SH) proceeded at the same rate, a competitive test was conducted to determine the selectivity of TCT in the presence of all the three nucleophiles at 0 °C for 30 min in a one-pot reaction (Figure 8). It was observed that NH is prevalent over SH and OH when present in the same reaction mixture, thereby indicating a high selectivity of the former. For the replacement of the second Cl atom, another equivalent of nucleophile was added in the presence of DIEA at rt overnight (12 h). Our results indicate that once NH is introduced, only another amine can be incorporated as either SH or OH, and cannot be incorporated further in m-DCTs under the same reaction conditions. The replacement of the third Cl atom was performed at 75 °C overnight. Of all the reactions attempted, the preferential order of incorporation was found to be first OH, then SH, and finally NH. However, the last replacement was still performed at a temperature incompatible with the application in peptides/proteins. Therefore, OH was replaced by phenol (pOH), as shown in Scheme 1 [9]. The first incorporation of pOH was achieved at −20 °C instead of 0 °C due to the high nucleophilicity of pOH. The presence of pOH also allowed the replacement of the third Cl atom by NH in 8 h at 35 °C (temperature compatible with applications using peptide/proteins).

**Scheme 3:**
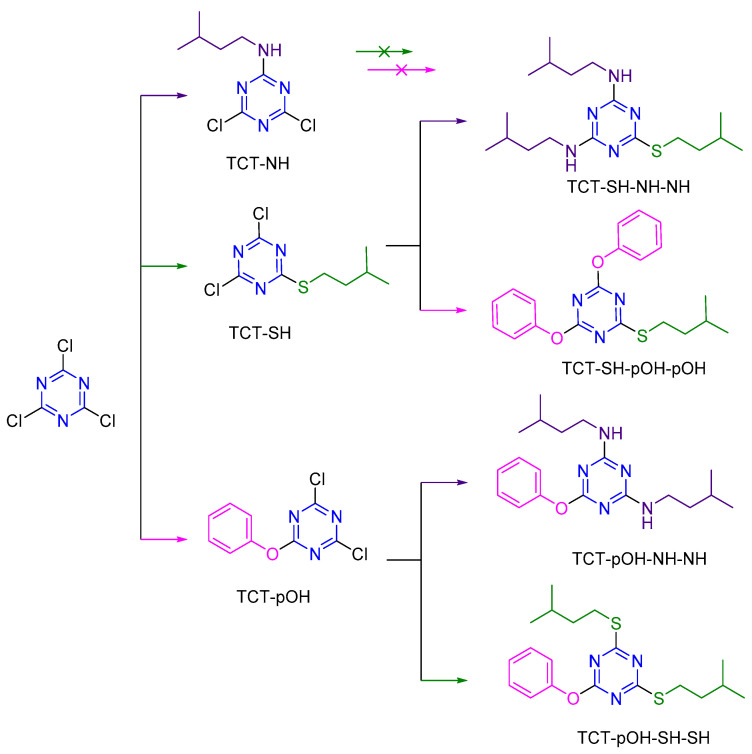
Reactions for the 1 + 2 mode between TCT and nucleophiles (NH, SH, and pOH).

With the advantage of pOH in modulating the reaction of the s-triazine core, a 1 + 2 mode (one nucleophile as the first substitution followed by another nucleophile for the other two positions) as an application for dendrimer synthesis was also attempted (Scheme 3).

m-DCTs were prepared as explained above, followed by the addition of 2 eq. of each nucleophile in the presence of DIEA. The reaction was stirred at 35 °C for 12 h. As depicted in Scheme 3, TCT-NH did not react with SH or pOH. These results are consistent with our earlier findings. For the rest, TCT-SH and TCT-pOH form the respective products, affording TCT-SH-(NH)_2_/TCT-SH-(pOH)_2_ and TCT-pOH-(NH)_2_/TCT-pOH-(SH)_2_, respectively. However, since the reactivity of TCT for the last Cl atom substitution reaction depends upon the order of nucleophile incorporation, its use as a linker is limited.

### 4.2. Tri-orthogonal Chemoselectivity with Azide as Modifier

The concept of Orthogonal Chemoselectivity using TCT as the core linker and amine (NH), thiol (SH), and alcohol/phenol (OH/pOH) as nucleophiles (mimicking amino acid side chains present in proteins) has been demonstrated [8,9]. Scheme 2 shows the series of reactions attempted to explain the order of incorporation.

**Scheme 4:**
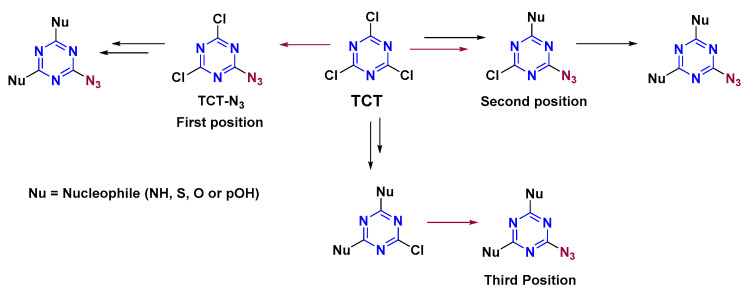
General scheme showing azide as modifier in all the three positions in TCT.

Any electron-withdrawing group tends to maintain the electron density in TCT reactivity. In this regard, azide was used as a modifier. Tri-orthogonal chemoselectivity was explained using the TCT core and the above-mentioned nucleophiles with N_3_ as a modifier. The presence of N_3_ in TCT was studied in all three positions (Scheme 4). TCT-N_3_ contains N_3_ in the first position. Few studies have addressed TCT-N_3_ [34,35,36]. The high reactivity of N_3_ makes it difficult to control the reaction selectively for the first position. Furthermore, due to the explosive nature of TCT-N_3_, scaling up and purification makes the process cumbersome. We have developed a safe method with high selectivity for the first position [16]. During the synthesis, an equimolar solution of NaN_3_ was added dropwise into a TCT solution in acetone. After 30 min, acetone was removed at 0 °C (to avoid unreacted NaN_3_ for further reaction). The aqueous solution was extracted using cold DCM (to avoid explosion in a bigger reaction scale due to generation of heat in the separatory funnel) and concentrated to obtain the crude product, which was purified using *n*-hexane in an isocratic silica gel column, to achieve an overall yield of 90.4% (4.7 g) [16].

**Scheme 5:**
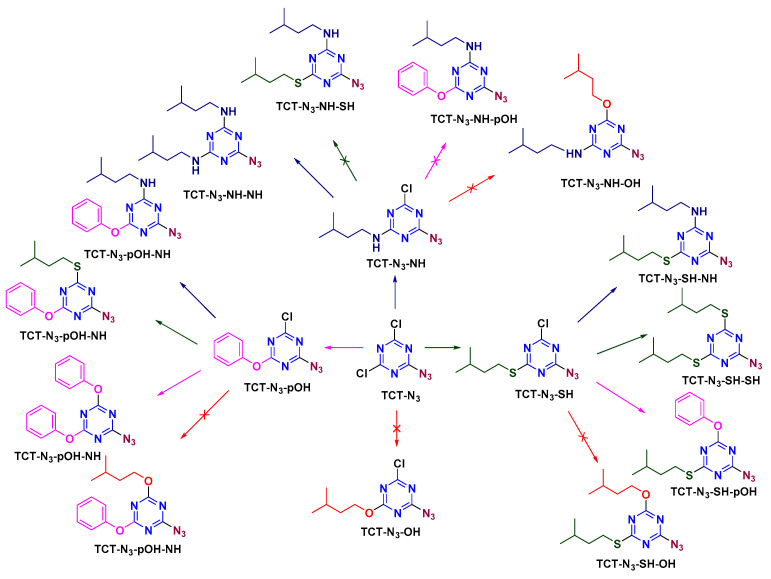
Azide as modifier in the first position on TCT.

Once TCT-N_3_ had been synthesized, the second Cl atom was replaced using NH, SH, and OH/pOH (Scheme 5). The reaction was completed in 30 min at 0 °C in the case of NH and SH, and at −20 °C for pOH, yielding TCT-N_3_-NH, TCT-N_3_-SH, and TCT-N_3_-pOH, respectively. In the case of TCT-N_3_-NH, only another NH can be used to form TCT-N_3_-NH-NH. The reaction was carried out at rt for 12 h. Regarding TCT-N_3_-SH/pOH, all the nucleophiles, except for OH, replaced the third Cl atom in the s-triazine ring. Given these observations, it was inferred that once N_3_ is present on TCT, OH cannot enter any position.

**Scheme 6:**
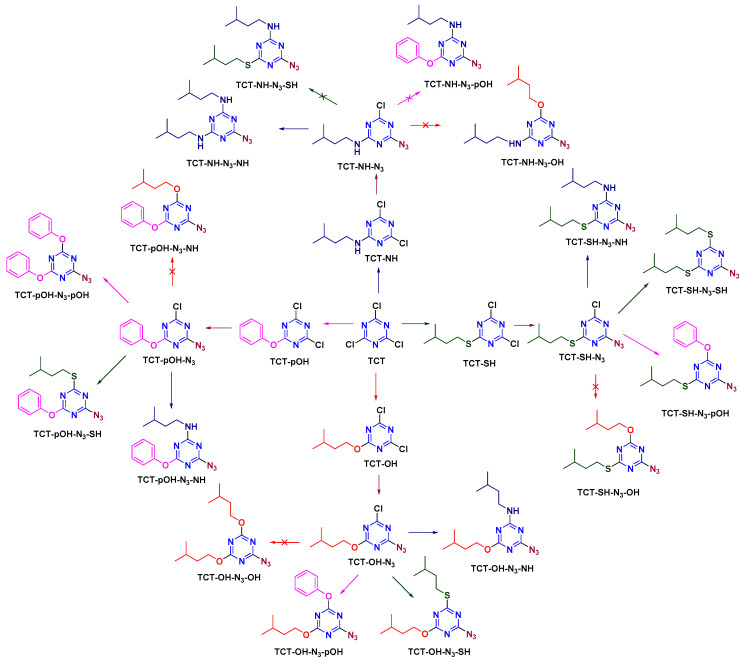
Azide as modifier in the second position on TCT.

Once N_3_ had been analyzed at the first position, its effect was evaluated in the second position (Scheme 6). m-DCTs were synthesized as explained above using NH, SH, and OH/pOH. Sodium azide (NaN_3_) was reacted with m-DCTs at 0 °C for 30 min in the presence of DIEA to afford d-MCTs (TCT-NH-N_3_, TCT-SH-N_3_, and TCT-OH/pOH-N_3_). TCT-OH-N_3_ synthesis was successful in this case, as N_3_ entered the s-triazine ring after OH had been incorporated (unlike the previous case). d-MCTs were then reacted with the above-mentioned nucleophiles at rt for 12 h. TCT-NH-N_3_ afforded only TCT-NH-N_3_-NH, as once an amine entered no other nucleophile other than another amine can react. TCT-SH-N_3_, TCT-OH-N_3_, and TCT-pOH-N_3_ afforded the respective trisubstituted s-triazine in a similar way (TCT-SH-N_3_-NH, TCT-SH-N_3_-SH, TCT-SH-N_3_-pOH, TCT-OH-N_3_-NH, TCT-OH-N_3_-SH, TCT-OH-N_3_-pOH, TCT-pOH-N_3_-NH, TCT-pOH-N_3_-SH, TCT-pOH-N_3_-pOH), except in the case of OH (as once N_3_ was incorporated in TCT, OH did not react).

**Scheme 7:**
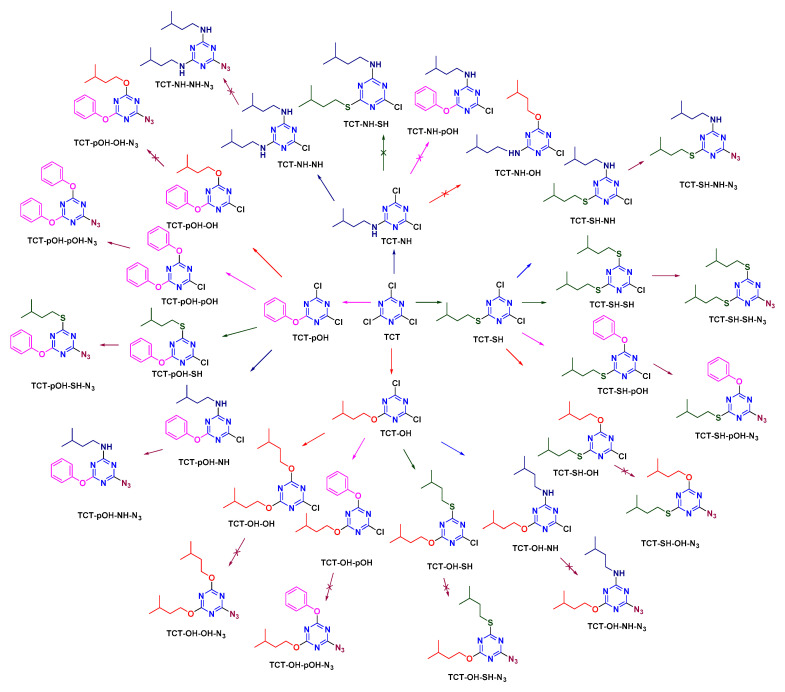
Azide as modifier in the third position on TCT.

The reactivity of N_3_ was then studied at the third position (Scheme 7). d-MCTs, where two substituents were either of the above-mentioned nucleophiles (NH, SH, OH, and pOH), were reacted with NaN_3_ at 40 °C for 12 h. The incorporation of N_3_ was observed in all cases, except when OH was present and in the case of d-MCTs with two NH.

**Scheme 8:**
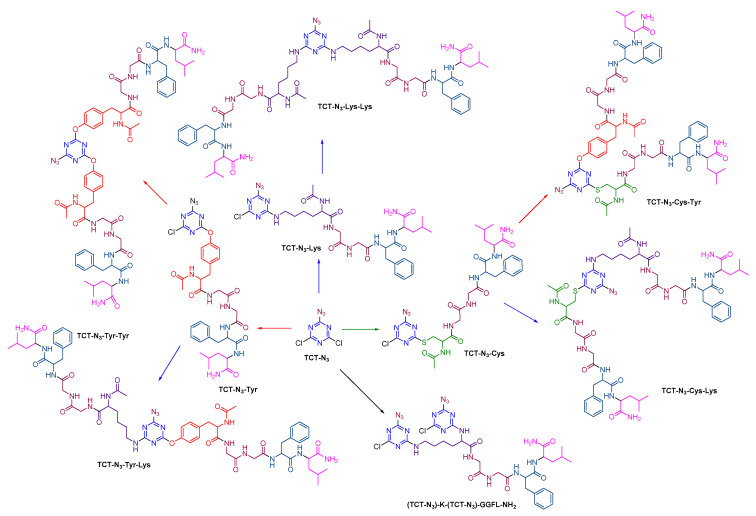
Sequential incorporation of pentapeptides onto TCT-N_3_.

With the overall idea that N_3_ in the first position was successful, we further explored its reactivity with peptides bearing NH, SH, and pOH. In this regard, pentapeptides (Ac-Xaa-Gly-Gly-Phe-Leu-NH_2_ where Xaa = Lys/Cys/Tyr) were sequentially incorporated into TCT-N_3_ as shown in Scheme 8. Ser peptide (Ac-Ser-Gly-Gly-Phe-Leu-NH_2_) was eliminated due to the poor nucleophilicity of the alcohol of Ser.

The first incorporation of peptide was performed using an equimolar solution of TCT-N_3_ and peptide at 0 °C for 30 min in the presence of K_2_CO_3_ (pH > 8) as base, using H_2_O:ACN (2:1) as the solvent mixture. The reaction mixture was extracted using ethyl acetate (to remove any unreacted TCT-N_3_) to afford pure TCT-N_3_-Lys, TCT-N_3_-Cys, and TCT-N_3_-Tyr. With the high selectivity of NH, as demonstrated earlier, TCT-N_3_-Lys was reacted with an equivalent of Lys peptide using K_2_CO_3_ as base at rt. The reaction was completed in 4 days, as monitored by HPLC. TCT-N_3_-Cys and TCT-N_3_-Tyr were reacted with Lys and Tyr under the same conditions. TCT-N_3_-Cys-Lys and TCT-N_3_-Cys-Tyr formed in 2 days at rt. TCT-N_3_-Tyr-Lys and TCT-N_3_-Tyr-Tyr formed at rt in 4 h and 8 h, respectively. The reactivity of TCT-N_3_ with α and ε NH_2_ of Lys in H-KGGFL-NH_2_ was also studied (Scheme 7). The different nucleophilicity of α and ε NH_2_ did not affect the incorporation order, as the reaction was observed to reach completion in 10 min at 0 °C, forming TCT-N_3_-K(TCT-N_3_)-GGFL-NH_2_, thereby demonstrating the high reactivity of amine toward TCT-N_3_.

## 5. Executive Summary

This report has two different parts. In the first one the biological activities of several libraries of s-triazine-based compounds is discussed. In the second part, different tips for the preparation of these substituted s-triazines based in the tri-orthogonal concept are covered.

Various s-triazine cores show therapeutic properties, including MAO-A and B inhibition, anticancer/ antiproliferative, and antileishmanial activity, and also antibacterial activity against MDR clinical isolates. They can also be used as drug nano-delivery systems. Some of the derivatives could be considered leads/hits for these targets. However, as in our example and the literature, the main nucleophile is amine (NH), and in some cases alcohol (OH).

The preferential order of incorporation of the different nucleophiles into the TCT core was found to be first alcohol, second thiol, and finally amine (but this order required a higher temperature especially for the replacement of the third Cl atom). Phenol (replacing alcohol) was used as a modifier in the TCT core, thereby removing the need for a higher temperature for the replacement of the third Cl atom (from reflux conditions to 35 °C). However, the applicability of phenol on the TCT core is restricted due to its limitation as the modifier for the first replacement.

Azide was another modifier used in TCT, and the tri-orthogonal chemoselectivity of the TCT core has been successfully demonstrated. Hence, the first replacement on TCT-N_3_ occurred at 0 °C, and the last one at rt. Competitive studies show that amine (NH) is highly selective in the presence of other nucleophiles, thereby making it a suitable linker for the protein/peptide conjugation. The use of azide as a modifier alongside two nucleophile (SH and pOH) substitutions can take place in any order at rt (a condition that is favorable for proteins/peptides).

## 6. Future Perspectives

This review reveals s-triazine as an excellent privileged heterocyclic core with a broad therapeutic application. Its privileged structure, related to the starting materials 1,3,5-trichloro-2,4,6-triazine (also known as cyanuric chloride), and its commercial availability, well-defined nature, low cost, and ubiquity in biological applications makes it a unique core for several applications.

The ability of TCT to undergo sequential nucleophilic substitution using regular nucleophiles (first Cl atom replacement at 0 °C, second at rt, and third at >90 °C) allows chemists to control the organic structure and make it react in the required condition for achieving each given objective. However, the last replacement at a higher temperature explains the poor diversity of TCT and limits its applicability. The use of TCT as a linker with modifiers will broaden the synthesis of derivatives under biologically favorable conditions in several medicinal chemistry projects.

Orthogonality and chemoselectivity are two concepts of modern organic chemistry that have been exploited in various fields of research, ranging from supramolecular to organic/bioconjugation chemistry. Although orthogonality is a subset of chemoselectivity, we have demonstrated the fusion of these two concepts as “Orthogonal Chemoselectivity” using TCT and defined this new concept as a “discrimination between reactive sites in any order”. The application of this concept in medicinal chemistry programs will allow for the preparation of a wide range of TCT derivatives in which other substituents can be present along with *N*-substitution, thereby broadening the applicability of TCT in this field.

In the current scenario, the loss of antibodies during the synthesis of Antibody Drug Conjugates (ADCs) occurs during disulfide cleavage for the bioconjugation of thiols with the linkers currently available [37,38]. ADCs also suffer from premature cleavage due to stability issues of the thioether bond [39]. With the successful fine-tuning of reaction conditions with modifiers, TCT-N_3_ can be exploited as a linker in the construction of Peptide Drug Conjugates/Antibody Drug Conjugates (PDCs/ADCs) [37,38,40]. The presence of several functionalities (amine, thiol, phenol) in peptides and proteins does not limit the use of the TCT core due to the high selectivity of amine in the presence of other nucleophiles, which opens the possibility of using them as linkers for protein/peptides bio-conjugation, with a further extension to antibodies.

## Data Availability

No new data were created or analyzed in this study. Data sharing is not applicable to this article.
